# Analysis of the *Wnt* gene repertoire in an onychophoran provides new insights into the evolution of segmentation

**DOI:** 10.1186/2041-9139-5-14

**Published:** 2014-04-03

**Authors:** Mattias Hogvall, Anna Schönauer, Graham E Budd, Alistair P McGregor, Nico Posnien, Ralf Janssen

**Affiliations:** 1 Department of Earth Sciences, Palaeobiology, Uppsala University, Villavägen 16, Uppsala, 75236, Sweden; 2 Department of Biological and Medical Sciences, Oxford Brookes University, Oxford, OX3 0BP, UK; 3 Department of Developmental Biology, Georg-August-University Göttingen, Justus-von-Liebig-Weg 11, Göttingen, 37077, Germany

**Keywords:** Development, Evolution, Segmentation, Segment polarity, *Wnt* signalling

## Abstract

**Background:**

The Onychophora are a probable sister group to Arthropoda, one of the most intensively studied animal phyla from a developmental perspective. Pioneering work on the fruit fly *Drosophila melanogaster* and subsequent investigation of other arthropods has revealed important roles for *Wnt* genes during many developmental processes in these animals.

**Results:**

We screened the embryonic transcriptome of the onychophoran *Euperipatoides kanangrensis* and found that at least 11 *Wnt* genes are expressed during embryogenesis. These genes represent 11 of the 13 known subfamilies of *Wnt* genes.

**Conclusions:**

Many onychophoran *Wnt* genes are expressed in segment polarity gene-like patterns, suggesting a general role for these ligands during segment regionalization, as has been described in arthropods. During early stages of development, *Wnt2*, *Wnt4*, and *Wnt5* are expressed in broad multiple segment-wide domains that are reminiscent of arthropod gap and *Hox* gene expression patterns, which suggests an early instructive role for *Wnt* genes during *E. kanangrensis* segmentation.

## Background

The phylum Onychophora is represented by only around 200 described species [1]. Like their probable sister group, the arthropods, onychophorans are segmented, a fact that is most obvious from the arrangement of up to 43 pairs of walking limbs on these animals. However, onychophorans differ from arthropods because they lack intersegmental ectodermal grooves, tagmosis is absent and their limbs are unsegmented [2]. Despite the great interest in, and growing understanding of, all aspects of arthropod biology, including the genetic regulation of segmentation (reviewed in, for example, [3-7]), relatively little is known about the onychophorans.

In *Drosophila melanogaster*, segmentation is under control of a hierarchic segmentation gene cascade that initially transforms aperiodic patterns of genetic information along the anterior-posterior body axis into a periodic pattern [8,9]. Comparative studies have revealed that at least some components of this hierarchical network are conserved and that the function of segment polarity genes in particular has been maintained during the evolution of arthropods and onychophorans [10-18]. The segment polarity genes act later in the hierarchy downstream of maternal effect genes, gap genes and pair rule genes, and regulate segment polarity and maintain segmental boundaries. The network of segment polarity genes includes morphogens, such as *Hedgehog* (*Hh*) and *Wingless* (*wg*/*Wnt1*).

The *Wnt* gene family comprises 13 subfamilies, of which 12 are found in protostomes, with *Wnt3* having been lost in the lineage leading to these animals [19,20]. Preliminary studies of the *Wnt* gene repertoire in arthropods suggest some lineages have lost one or more *Wnt* genes in the course of evolution (summarized in [20]): for example, *Wnt2* and *Wnt4* appear to have been lost in insects.

To further study the role that *Wnt* genes play in development and evolution, we surveyed the repertoire of these genes in the onychophoran *Euperipatoides kanangrensis* and investigated their expression during its embryogenesis. We found that at least 11 of the predicted 12 *Wnt* genes found in protostomes are expressed during onychophoran ontogenesis. Our data suggest that onychophoran *Wnt* genes are likely to be involved in segment border formation or maintenance, intrasegmental patterning and possibly even the determination of segment identity. The latter function would not only represent an onychophoran-specific feature of *Wnt* gene function, but also suggest a role for these genes in segmentation beyond that of segment regionalization.

## Methods

### Animal husbandry and embryo preparation

Female specimens of *E. kanangrensis* were collected in Kanangra-Boyd National Park, New South Wales, Australia. To obtain all developmental stages, we dissected developing embryos of various stages in the months from September to December. Each female carries up to 100 embryos, representing a series of developing stages (sometimes even ranging from the one-cell stage up to the fully developed embryo). The chorion and vitelline membrane were removed by hand with Dumont size 5 forceps and directly afterwards the embryos were fixed in 4% formaldehyde in 0.1 M phosphate-buffered saline with 0.1% Tween-20 (PBST) (pH 7.4) for four to six hours at room temperature. Embryos were then dehydrated stepwise into 100% methanol and stored at −20°C for at least three weeks before being used for *in-situ* hybridization experiments.

### PCR and gene cloning

RNA isolation and cDNA synthesis were described in [14]. Gene fragments of all *Wnt* gene orthologues described here were isolated by means of PCR with gene specific primers based on the sequences found in a sequenced embryonic transcriptome. For further information on the transcriptome see [18].

All *Wnt* gene fragments were cloned into the pCRII vector (Invitrogen, Carlsbad, CA, USA), and sequences were determined by means of Big Dye chemistry on an ABI3730XL analyzer by a commercial sequencing service (Macrogen, Amsterdam, The Netherlands). Sequences of the newly discovered *E. kanangrensis Wnt* genes are available from the EMBL nucleotide database under accession numbers HG529208 (*Wnt2*), HG529209 (*Wnt4*), HG529210 (*Wnt5*), HG529211 (*Wnt6*), HG529212 (*Wnt7*), HG529213 (*Wnt9*), HG529214 (*Wnt10*), HG529215 (*Wnt11*), HG529216 (*Wnt16*), and HG529217 (*WntA*).

### *In-situ* hybridization, cell nuclei staining and data documentation

*In-situ* hybridization experiments were performed as described previously [21]. Cell nuclei were stained with 1 μg/ml DAPI (4-6-diamidino-2-phenylindole) in PBST for 20 minutes followed by several washing steps in PBST. Embryos were analyzed under a Leica dissection microscope equipped with a Leica DC100 digital camera. Brightness, contrast and colour values were adjusted if necessary, using the image processing software Adobe Photoshop CS2 (Version 9.0.1 for Apple Macintosh).

### Phylogenetic analysis

The amino acid sequences of *E. kanangrensis Wnt* genes were aligned with those of *Wnt* sequence dataset 1 from [20] using T-Coffee followed by manual editing in SeaView [22,23].

Bayesian phylogenetic analyses were performed with MrBayes [24] using a fixed WAG amino acid substitution model with gamma-distributed rate variation across sites (with four rate categories). An unconstrained exponential prior probability distribution on branch lengths and an exponential prior for the gamma shape parameter for among-site rate variation was applied. The final topology was estimated using 1,100,000 cycles for the MCMCMC (metropolis-coupled Markov chain Monte Carlo) analysis with four chains and the chain-heating temperature set to 0.2. The Markov chain was sampled every 200 cycles. The starting trees for the chains were randomly selected. Clade support was assessed with posterior probabilities computed with MrBayes.

## Results

### The *Wnt* gene repertoire of *E. kanangrensis*

**Figure 1:**
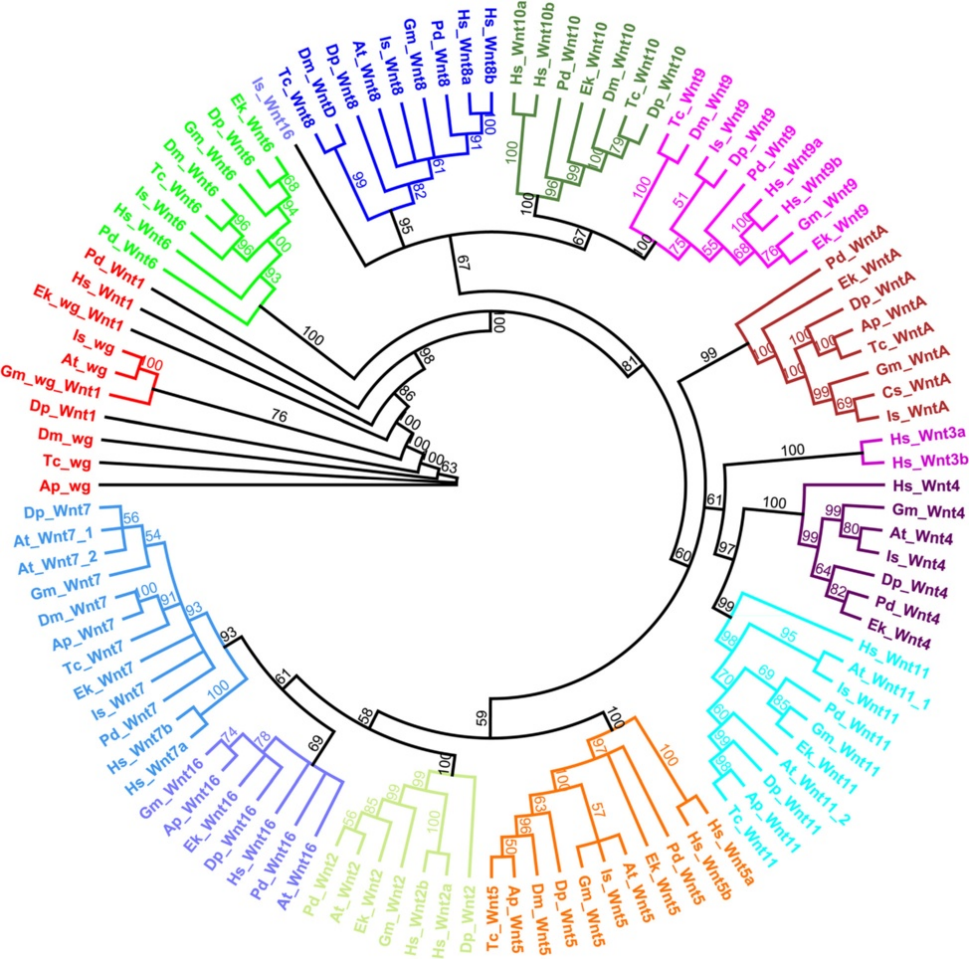
**Phylogenetic analysis of *Wnt *genes.** Maximum likelihood tree of Wnt amino acid sequences. The support value of each node is given as posterior probability (as a percentage) from the Bayesian analysis on the branches. Note that Is-Wnt16 is the only Wnt sequence that does not cluster with the respective orthologues. Included metazoan species are: *Parasteatoda tepidariorum* (At), *Acyrthosiphon pisum* (Ap), *Cupiennius salei* (Cs), *Daphnia pulex* (Dp), *Drosophila melanogaster* (Dm), *Euperipatoides kanangrensis* (Ek), *Glomeris marginata* (Gm), *Homo sapiens* (Hs), *Ixodes scapularis* (Is), *Platynereis dumerilii* (Pd) and *Tribolium castaneum* (Tc).

**Figure 2:**
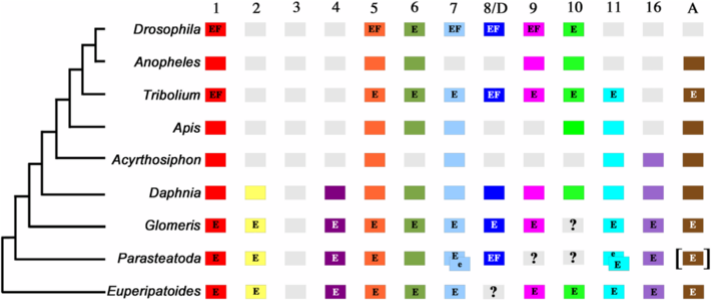
**Repertoire of *Wnt *subfamilies in arthropods, an onychophoran and a lophotrochozoan.** Grey boxes indicate putatively lost *Wnt* subfamilies. Question marks indicate *Wnts* that have not been found but cannot be defined as ‘missing’ because assigned genomes have not been sequenced or annotated. Duplicated *Wnts* are represented by overlapping boxes. Square brackets indicate that spider *WntA* was isolated from another spider, *C. salei*. E, embryonic expression patterns are available; e, embryonic expression has been studied but specific expression patterns could not be detected; F, functional data are available. Note that for *T. castaneum* all *Wnts* have been knocked down by means of RNAi, but phenotypes could only be detected for *wg/Wnt1* and *Wnt8/D. Wnt3* is present in deuterostomes and cnidarians, but has been lost in the lineage leading to the protostomes.

**Figure 3:**
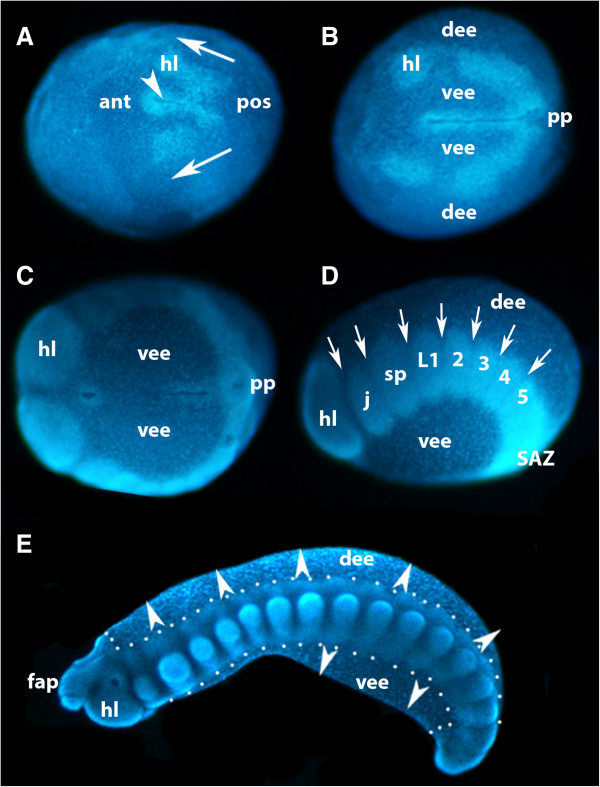
**Formation of the germ band in *E. kanangrensis.*** A germ disc appears after the blastoderm has formed (not shown; [31]), in the centre of which forms a slit-like furrow (arrowhead in A) defining the AP axis. Anterior is to the left. **(A)** Stage 2 to 3 embryo, ventral view. Cells invaginating at the posterior pole move on either side of the indentation towards anterior (arrows), forming a split germ band. **(B)** Stage 5 embryo, ventral view. Split germ band subsequently moves towards the anterior and first coelomic pouches bud off from otherwise uniform halves of germ band. The distance between the furrow and the halves of the germ band has increased and ventral extraembryonic ectoderm covers the area between furrow and germ band on either side of the furrow; dorsal to the halves of the germ band is dorsal extraembryonic ectoderm. **(C)** Stage 8 embryo, ventral view. At later stages, the anterior extent of the split germ band (future head lobes) meets and fuses anterior to the anterior pole of the furrow. **(D)** Embryo of **C**, lateral view; dorsal is up. Coelomic pouches are recognizable by areas of higher cell density. As with short germ arthropods, anterior segments represent older (more developed) segments. In the posterior part of the split germ band (anterior to SAZ), coelomic pouches have not yet segregated from newly formed tissue. **(E)** Stage 15 embryo, lateral view; outline of embryo proper marked by dots. Dorsal and ventral embryonic tissue begins to grow out (arrowheads). 2 to 5, second to fifth walking-limb-bearing segment; ant, anterior; AP, anterior-posterior; dee, dorsal extraembryonic ectoderm; fap, frontal appendage; hl, head lobe; j, jaw; L1, first walking-limb-bearing segment; pos, posterior; pp, posterior pit region; SAZ, segment addition zone; sp, slime papilla; vee, ventral extraembryonic ectoderm.

Phylogenetic analysis of *E. kanangrensis* Wnt amino acid sequences shows that this onychophoran has at least 11 *Wnt* genes representing the *Wnt1*, *Wnt2*, *Wnt4*, *Wnt5*, *Wnt6*, *Wnt7*, *Wnt9*, *Wnt10*, *Wnt11*, *Wnt16* and *WntA* subfamilies (Figures 1 and 2). Therefore, *E. kanangrensis* has all *Wnt* genes reported in protostomes and arthropods except *Wnt8* (Figure 1). To investigate the potential roles of these genes in comparison with other animals, we then examined their expression in *E. kanangrensis* embryos (see Figure 3 for an overview of early embryogenesis in this onychophoran).

### Expression of *E. kanangrensis wg/Wnt1*

The expression of the *E. kanangrensis wingless* (*wg/Wnt1*) orthologue has been described previously [17]. It is expressed like a typical segment polarity gene in transverse stripes in the middle of each segment and anterior to the expression of *engrailed* (*en*) and *hedgehog* (*hh*) [18], as well as in the tips of all developing appendages [17].

### Expression of *E. kanangrensis Wnt2*

**Figure 4:**
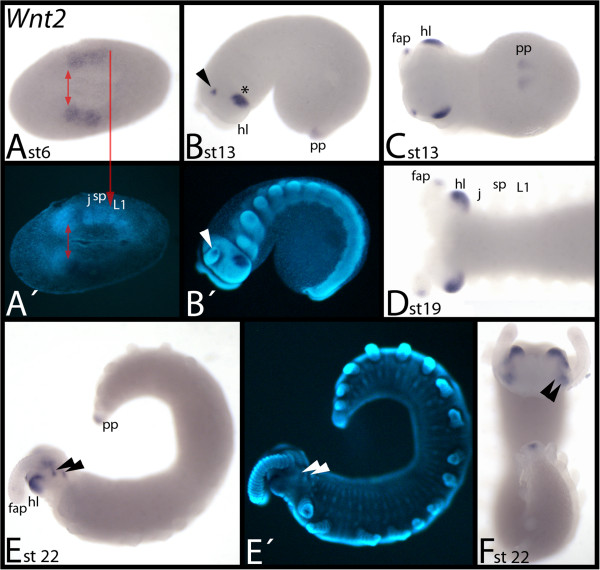
**Expression of *E. kanangrensis Wnt2*.** In all panels except **(F)** (anterior up), anterior is to the left. **(A)** Ventral view. Red double-arrow marks the anterior border of expression. Red arrow marks posterior border of expression. **(B)** Lateral view. Arrow points to expression in the tip of the frontal appendage. **(C)** Ventral view of embryo of **(B). (D)** Ventral view of the anterior of an embryo. **(E)** Lateral view. Arrowheads indicate dot-like expression posterior to the base of the frontal appendages. **(F)** Dorsal view. Embryo of **(E)**. Arrowheads as in E. **(A**′**)**, **(B**′**)** and **(E**′**)** represent DAPI staining of the embryos of **(A)**, **(B)** and **(E)**. fap, frontal appendage; hl, head lobe; j, jaw; L1, first walking-limb-bearing segment; pp, posterior pit region; sp, slime papilla; st, stage.

During early embryogenesis in *E. kanangrensis*, *Wnt2* is expressed in broad domains covering the posterior of the future head lobes, and the primordia of the jaws and slime papillae-bearing segments (Figure 4A). At later stages, it is concentrated in the tips of the frontal appendages, the head lobes and in the posterior pit (Figure 4B-F). The expression in the head lobes is lateral and below the eyes. Expression in the tips of the frontal appendages is strong at first but weakens in older stages until it disappears in the oldest investigated stages (Figure 4B-F). Late during ontogenesis, additional expression appears on the dorsal side of the head lobes near the bases of the frontal appendages (Figure 4E, F).

### Expression of *E. kanangrensis Wnt4*

**Figure 5:**
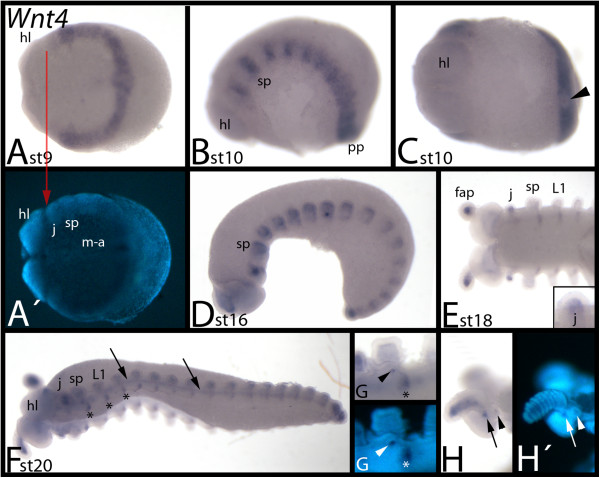
**Expression of *E. kanangrensis Wnt4.*** Anterior is to the left. **(A)** Ventral view. Red arrow marks the anterior border of expression. **(B)** Lateral view. **(C)** Embryo of **(B)**, ventral view. Arrowhead indicates lack of expression in the posterior pit region. **(D)** Lateral view. **(E)** Anterior portion of embryo and close-up of jaw. Note that expression in the jaw is only in the mesoderm. **(F)** Ventrolateral view. Arrows indicate expression between the bases of the limbs. Asterisks mark expression in the ventral nervous system. **(G)** Embryo of **(F)**. Close-up of slime-papillae-bearing segment. Arrowhead indicates staining in salivary gland. Asterisk marks expression in the ventral nervous system. **(H)** Embryo of **(F)**. Close-up of head. Arrow and arrowhead indicate dot-like expression at base of frontal appendage. Note that expression in the frontal appendage is mesodermal. **(A′)**, **(G′)** and **(H′)** represent DAPI staining of the embryos of **(A)**, **(G)** and **(H)**. fap, frontal appendage; hl, head lobe; j, jaw; L1, first walking-limb-bearing segment; m-a, mouth-anus furrow; pp, posterior pit region; sp, slime papilla; st, stage.

*Wnt4* is first expressed ubiquitously in the posterior segments, including the posterior segment addition zone (SAZ) (Figure 5A); but the posterior pit region is free of transcripts (shown for a slightly older embryo in Figure 5C). There is also a sharp anterior expression boundary between the head lobes and the jaw-bearing segment (Figure 5A), and early expression in the jaw-bearing segment is weaker (Figure 5A, B). During subsequent stages, this band of expression transforms into a segmental pattern (Figure 5B). When the limbs form, *Wnt4* is clearly expressed in the mesoderm of the frontal appendages (Figure 5D, E) and jaws (Figure 5E, F). However, the slime papillae and walking limbs do not express *Wnt4*. At later developmental stages, *Wnt4* expression appears stepwise in a segmental pattern from anterior to posterior in a position ventral and posterior to the bases of the jaw, the slime papillae and the walking-limb-bearing segments (Figure 5E). This expression persists during later developmental stages (Figure 5F). At this point, a faint anterior-to-posterior stripe of expression appears ventral to the limbs (Figure 5F). The tissue in the developing openings to the salivary glands possibly expresses *Wnt4* at late stages (Figure 5G); this, however, may represent nonspecific staining that sometimes occurs in this structure during late developmental stages. At stage 20, two spots of expression appear in each hemisphere of the head lobes posterior to the bases of the frontal appendages (Figure 5H).

### Expression of *E. kanangrensis Wnt5*

**Figure 6:**
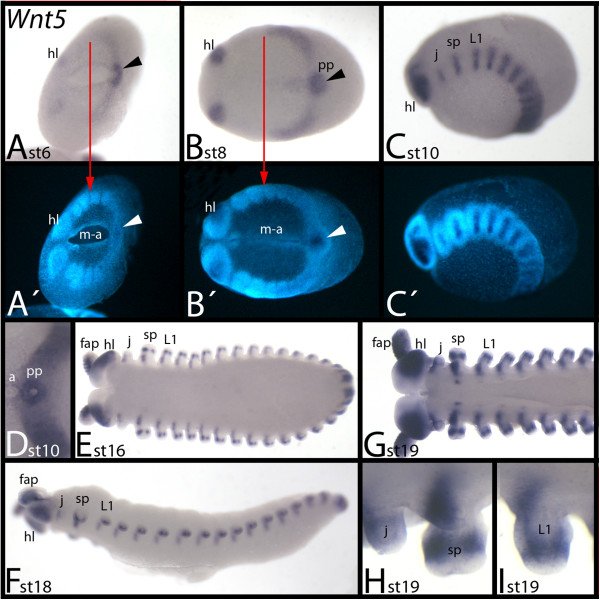
**Expression of *E. kanangrensis Wnt5.*** Anterior is to the left. **(A)** Ventral view. Red arrow marks the anterior border of expression (of the broad domain; note additional expression in the head lobes). Arrowhead indicates strong expression in the posterior pit region. **(B)** Ventral view. Arrow and arrowhead as in **(A). (C)** Lateral view. **(D)** Ventral view. Close-up of the posterior of the embryo of **(C)**. Note the strong expression in a ring around the centre of the posterior pit region. **(E)** Ventral view. **(G)** Ventral view. Anterior part of an embryo. **(F)** Lateral view. **(H)** Close-up of a jaw and a slime papilla. **(I)** Close-up of a walking limb. **(A′)**, **(B′)** and **(C′)** represent DAPI staining of the embryos of **(A)**, **(B)** and **(C)**. a, anus; fap, frontal appendage; hl, head lobe; j, jaw; L1, first walking-limb-bearing segment; m-a, mouth-anus furrow; pp, posterior pit region; sp, slime papilla; st, stage.

At early developmental stages, *Wnt5* is expressed in the head lobes and posterior of the embryo with a sharp border between the slime-papillae-bearing segment and the first walking-limb-bearing segment (Figure 6A, B). At subsequent stages, the levels of expression increase in all *Wnt5*-expressing tissues, except the posterior pit. *Wnt5* expression encircles the posterior pit and also extends towards the anterior into the posterior portion of the mouth-anus furrow but more weakly (Figure 6B). At around stage 10, *Wnt5* is expressed in transverse stripes in the middle of each segment (Figure 6C). At this point, *Wnt5* forms a ring of expression around the centre of the posterior pit (Figure 6D). At later stages, expression is in the anterior portion of the frontal appendages, jaws, slime papillae and walking limbs (Figure 6E-I). Expression, however, is restricted to the proximal region of the slime papillae and walking limbs, and for all segments this domain reaches into the tissue ventral to the bases of the limbs where the ventral nervous system will form. At around stage 19, spots of segmental expression appear ventral to the previously described expression; these spots appear in an anterior-to-posterior order, with the most anterior domains being located in the slime-papillae-bearing segments (Figure 6G). In addition to expression in the limbs described, *Wnt5* is also expressed in a median ring in the slime papillae and the walking limbs (Figure 6E, G-I). Throughout development, a broad ventral and lateral domain of expression is also observed in each hemisphere of the head lobes.

### Expression of *E. kanangrensis Wnt6*

**Figure 7:**
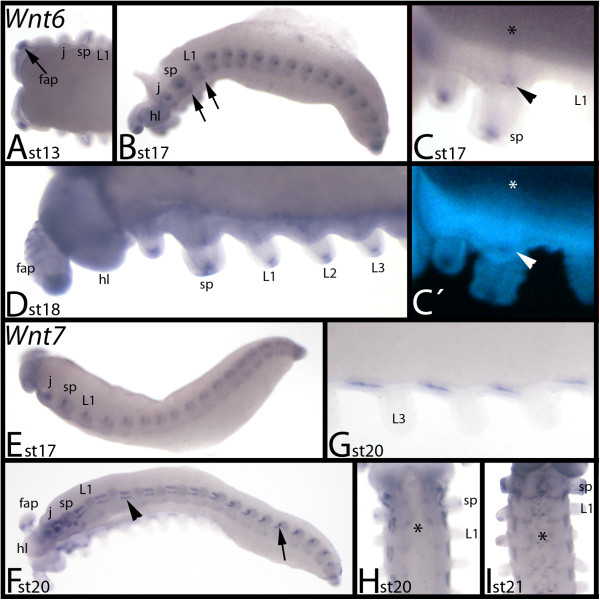
**Expression of *E. kanangrensis Wnt6 *and *Wnt7. *(A-D)** Expression of *Wnt6*; **(E-I)** Expression of *Wnt7*. In **(A-G)**, anterior is to the left; in **(H)** and **(I)**, anterior is up. **(A)** Dorsal view. Arrow indicates expression in the tip of the developing frontal appendage. **(B)** Lateral view. Arrows indicate expression in the ventral nervous system. **(C)** Ventral view. Close-up of the jaw- and slime papillae-bearing segments. Arrow indicates staining in the salivary gland. Asterisk marks transverse ventral stripe. Note that it is level with the salivary gland. **(D)** Ventral view. Close-up of the anterior part of an embryo. **(E)** Lateral view. **(F)** Ventrolateral view. Arrowhead indicates expression between the limbs. Arrow indicates the same expression in a younger (more posterior) segment. Note the transformation of this expression domain from younger towards older segments. **(G)** Close-up of segmentally reiterated mesodermal expression in the trunk. **(H)** Ventral view. Asterisk marks expression in the ventral nervous system. **(I)** Ventral view. Asterisk marks expression in the ventral nervous system. **(C′)** represents DAPI staining of the embryo of **(C)**. fap, frontal appendage; hl, head lobe; j, jaw; L1 to L3, first to third walking-limb-bearing segments; sp, slime papilla; st, stage.

*Wnt6* is not expressed before limb bud growth is observed (not shown). The first detectable expression is evident at stage 13, in the tips of the frontal appendages (Figure 7A). At later stages, expression appears in the tips of all other limbs (Figure 7B-D), and as very faint transverse segmental stripes in the centre of each segment (Figure 7B, C).

### Expression of *E. kanangrensis Wnt7*

Expression of *Wnt7* is also absent from early developmental stages (not shown). The earliest expression is observed at the bases of all the appendages of the trunk segments (Figure 7E). At subsequent stages, this expression becomes stronger and eventually transforms into a series of short longitudinal stripes in the mesoderm between the bases of the limbs along the anterior-posterior axis of the embryo (Figure 7F, G). During late stages, segmental expression appears in the developing ventral nervous system (Figure 7H, I).

### Expression of *E. kanangrensis Wnt9*

**Figure 8:**
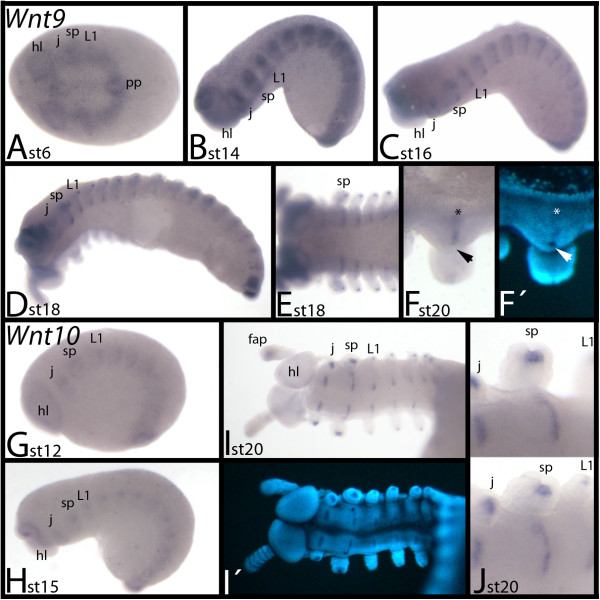
**Expression of *E. kanangrensis Wnt9 *and *Wnt10*. (A-F)** Expression of *Wnt9*. **(G-J)** Expression of *Wnt10*. Anterior is to the left. **(A)** Ventral view. Ubiquitous expression. **(B**) Lateral view. **(C)** Lateral view. **(D)** Ventrolateral view. **(E)** Ventral view. Anterior part of an embryo. **(F)** Ventral view. Close-up of the slime-papillae-bearing segment. Note the transverse stripe of expression (asterisk) that is level with the opening of the salivary gland (arrowhead) (cf. **F**′). **(G)** Lateral view. **(H)** Lateral view. **(I)** Ventral view, focused on the anterior of the embryo. **(J)** View of two slightly different angles of the same area of the embryo showing the slime-papillae-bearing segment. Note that the transverse stripe of expression is level with the posterior region of the salivary gland opening. **(F′)** and **(I′)** represent DAPI staining of the embryos of **(F)** and **(I)**. fap, frontal appendage; hl, head lobe; j, jaw; L1, first walking-limb-bearing segment; pp, posterior pit region; sp, slime papilla; st, stage.

During early stages, *Wnt9* is ubiquitously expressed (Figure 8A). Later, segmental expression is observed, which transforms into transverse stripes in the centre of the segments (Figure 8B, C). Subsequently, *Wnt9* is also expressed in the tips of the limbs (except for the frontal appendages, in which *Wnt9* is expressed throughout the mesoderm) (Figure 8D, E). In the slime-papillae-bearing segment *Wnt9* expression marks the position of the salivary gland openings (Figure 8F).

### Expression of *E. kanangrensis Wnt10*

During early stages, *Wnt10* is weakly expressed throughout *E. kanangrensis* embryos (not shown). Later, differential expression appears in the tips of the limb buds and as transverse stripes in the centre of the trunk segments (Figure 8G-J). However, expression in the tips of the frontal appendages is weaker than in the other appendages (Figure 8I). Segmental stripes of expression are located posteriorly adjacent to the openings of the salivary glands, which also express *Wnt10* (Figure 8J).

### Expression of *E. kanangrensis Wnt11*

**Figure 9:**
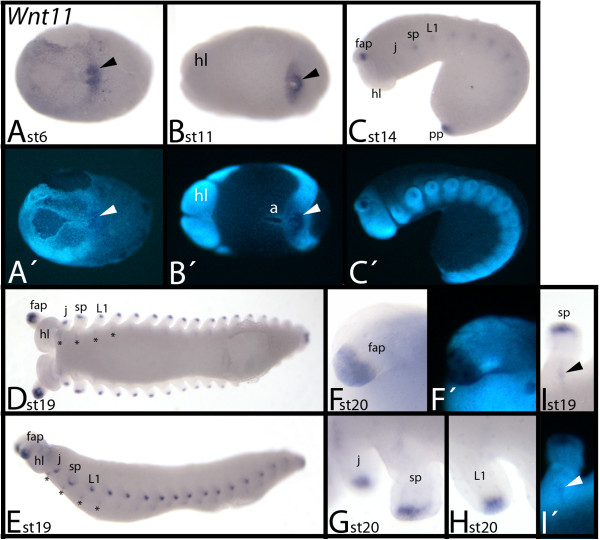
**Expression of *E. kanangrensis Wnt11.*** Anterior is to the left. **(A)** Ventral view. Arrow indicates strong expression in the posterior pit region. **(B)** Ventral view. Arrow indicates strong expression in the posterior pit region. **(C)** Lateral view. **(D)** Ventral view. Asterisks mark upcoming expression in segmental transverse stripes. **(E)** Embryo of (D), lateral view. Asterisks mark upcoming expression in segmental transverse stripes. **(F)** Close-up of frontal appendage. **(G)** Close-up of a jaw and a slime papilla. **(H)** Close-up of a walking limb. **(I)** Close-up of a slime-papillae-bearing segment, focused on the transverse stripe of expression. Note that this stripe is level with the opening of the salivary gland (arrowhead; cf. **I**′). **(A′)**, **(B′)**, **(C′)**, **(F′)** and **(I′)** represent DAPI staining of the embryos of **(A)**, **(B)**, **(C)**, **(F)** and **(I)**. a, anus; fap, frontal appendage; hl, head lobe; j, jaw; L1, first walking-limb-bearing segment; pp, posterior pit region; sp, slime papilla; st, stage.

During early developmental stages, *Wnt11* expression is detectable in the posterior pit region (Figure 9A). However, the indentation of the posterior pit does not express *Wnt11*, and expression does not extend into the future anus (Figure 9A, B). Later, expression appears in the tips of all appendages (Figure 9C). This expression persists throughout embryogenesis (Figure 9D-H). At stage 19, faint expression in segmental stripes appears in an anterior-to-posterior order in all trunk segments (Figure 9D, E, I).

### Expression of *E. kanangrensis Wnt16*

**Figure 10:**
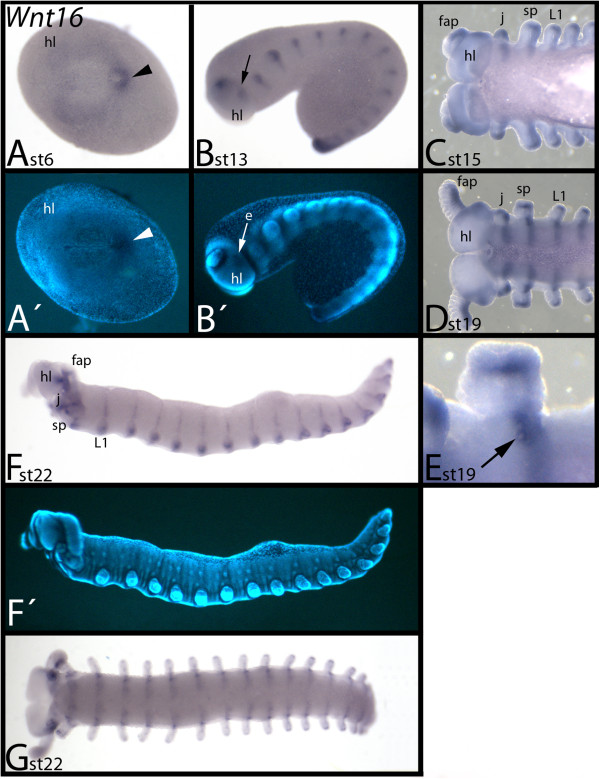
**Expression of *E. kanangrensis Wnt16.*** Anterior is to the left. **(A)** Ventral view. Arrowhead indicates expression in the posterior pit region. **(B)** Lateral view. Arrow indicates expression posterior to the eye groove. **(C)** Ventral view. Anterior part of an embryo. **(D)** Ventral view. Anterior part of an embryo. **(E)** Close-up of a slime papillae-bearing segment. Note that the anterior border of the segmental stripe is level with the opening of the salivary gland (arrow). **(F)** Lateral view. Note the segmental stripes extending into tissue dorsal to the limbs. **(G)** Embryo of **(F)**, ventral view. **(A′)**, **(B′)** and **(F′)** represent DAPI staining of the embryos of **(A)**, **(B)** and **(F)**. e, eye groove; fap, frontal appendage; hl, head lobe; j, jaw; L1, first walking-limb-bearing segment; sp, slime papilla; st, stage.

In early embryos, *Wnt16* is expressed around the posterior pit region, and weakly but ubiquitously in all other tissue except the mouth-anus furrow (Figure 10A). During later stages, expression appears in the base of the frontal appendages, a more posterior region adjacent to the eye grooves and, in a segmental pattern, the trunk (Figure 10B). The segmental pattern then transforms into transverse stripes in dorsal (dorsal to the position of the limbs) and ventral (ventral to the position of the limbs) tissue (Figure 10C-G). This is different from the expression of other *E. kanangrensis Wnt* genes, which are expressed as stripes exclusively in ventral, but not dorsal, tissue. The ventral transverse stripes are broader than the comparable stripes of other *Wnt* genes and they cover the complete posterior compartment of the segments, including the openings of the salivary glands in the slime-papillae-bearing segments (Figure 10E). *Wnt16* is also expressed in posterior tissue of the limbs and in the tips of the limbs (Figure 10C-E).

### Expression of *E. kanangrensis WntA*

**Figure 11:**
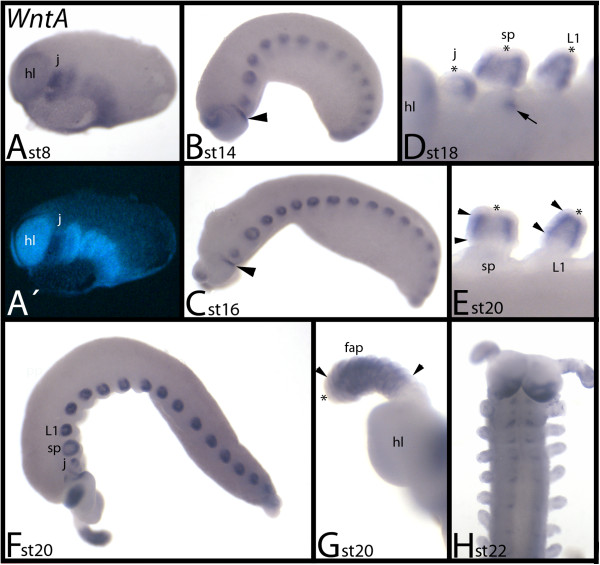
**Expression of *E. kanangrensis WntA.*** In all panels (except for panel **H**, where anterior is up), anterior is to the left. **(A)** Ventrolateral view. **(B)** Lateral view. Arrowhead indicates expression at the posterior rim of the head lobe. **(C)** Lateral view. Arrowhead indicates expression at the posterior rim of the head lobe. **(D)** Close-up of the anterior appendages. Asterisks mark tips of jaw, slime papilla and walking limb that are free from expression. Arrow indicates staining in the opening of the salivary gland. **(E)** Close-up of slime papilla and walking limb of an older embryo (compare with **D**). Arrowheads mark anterior and posterior border of expression in the limbs. Asterisks mark tips of jaw, slime papilla and walking limb that are free from expression. **(F)** Lateral view. **(G)** Close-up of frontal appendage and head lobe. Arrowheads mark anterior and posterior border of expression in the limbs. Asterisks mark tips of jaw, slime papilla and walking limb that are free from expression. (**H)** Ventral view. Note developing expression in the ventral nervous system. **(A**′**)** represents DAPI staining of the embryo of **(A)**. fap, frontal appendage; hl, head lobe; j, jaw; L1, first walking-limb-bearing segment; sp, slime papilla; st, stage.

During early developmental stages, *WntA* is ubiquitously expressed, with the strongest expression observed in the jaw-bearing segment (Figure 11A). At later stages, it is expressed in a broad leg-gap-gene-like domain in all limbs (Figure 11B-G) (compare with expression of onychophoran limb gap genes in [20]). Only the tips of the limbs and the most proximal region do not express *WntA*. In the jaws, *WntA* is expressed in a posterior and central region (Figure 11D). The posterior rim of the head lobes expresses *WntA* (Figure 11B-D, F, H). During later stages, the openings of the salivary glands also exhibit expression (but this may represent an unspecific signal) (Figure 11D). *WntA* is only weakly expressed in the SAZ. At around stage 22, segmental expression appears in the ventral nervous system, and the domain of expression in the head lobes is enlarged (Figure 11H).

## Discussion

### *Wnt* genes and segment identity

**Figure 12:**
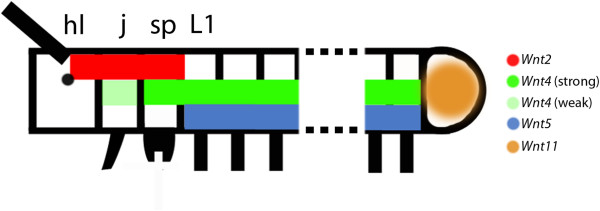
**Gap-gene-like expression patterns.** Depicted are the anterior and posterior parts of an embryo. Dashed lines indicate missing segments. Broad early multi-segment-wide domains of expression of *Wnt* genes are represented by coloured bars. hl, head lobe; j, jaw; L1, first walking-limb-bearing segment; sp, slime papilla.

We observed that *Wnt2*, *Wnt4*, *Wnt5* and *Wnt11* are expressed in broad, several-segment-wide, domains with distinct anterior boundaries, and in the case of *Wnt2* also a clear posterior boundary (Figure 12). These expression patterns are reminiscent of that of onychophoran and (to some extent) arthropod gap and *Hox* genes (Additional file 1: Figure S1) [25-28]. In particular, the early expression of *Wnt2* is similar to that of anterior *Hox* genes and gap genes in arthropods, which provide each segment with its specific identity (summarized in [29,30]). The early expression patterns of *E. kanangrensis Wnt* genes thus suggest a possible role in providing segmental identity. Such a possible function, however, must be different from *Hox* and gap genes, which act as direct selector genes on the segments’ identity. The *Wnt* genes may act downstream of initial selector genes, such as the *Hox* genes and other hitherto undetected genes that act anterior to the *Hox* genes during development of the anterior body of onychophorans. Moreover, while the expression of onychophoran *Hox* genes is restricted to the slime-papillae-bearing segment and backwards, the anterior expression borders of two *Wnt* genes, *Wnt2* and *Wnt4*, is located more anteriorly (Figure 12 and Additional file 1: Figure S1). *Wnt* genes may thus contribute to defining or maintaining segment identity of the walking-limb-bearing segments (expressing *Wnt4* and *Wnt5*), the slime-papillae-bearing segment (expressing *Wnt2* and *Wnt4*), and the jaw-bearing segment (expressing *Wnt2* and *Wnt4* (weakly)). The posterior region of the head lobes only expresses *Wnt2* and the anterior portion of the head lobes is free of any *Wnt* gene expression.

Since the composition of the anterior head in onychophorans is still uncertain, the anterior border of *Wnt2* expression within the head lobes indirectly raises the question of whether the head lobes represent two segments (or at least two independently patterned regions). So far, only one coelomic pouch has been identified in the developing head lobes (for example, [31]), and only one transverse stripe of *hedgehog* (*hh*) expression lies at the posterior rim of the head lobe [18].

### *Wnt* genes in segmentation and segment regionalization

The arthropod *wingless/Wnt1* (*wg/Wnt1*) gene is a classical segment polarity gene that is involved in maintaining segmental boundaries and intrasegmental patterning (reviewed in [32-34]). In *D. melanogaster*, other arthropods, and onychophorans, *wg* is expressed anterior adjacent to the expression of *engrailed* (*en*) (for example, [35,36,14,17]).

**Figure 13:**
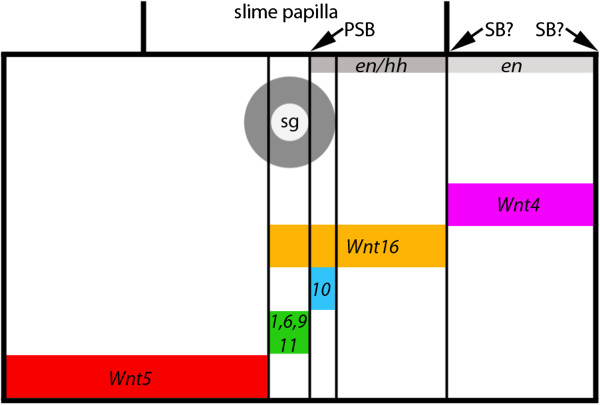
**Segment polarity gene-like intrasegmental expression.** The slime-papillae-bearing segment is represented (broad black lines) in embryos older than stage 10. The positions of the slime papillae are indicated by transverse broad black lines. Thin black lines mark intrasegmental borders of *Wnt* gene expression. The grey circle marks the position of the salivary gland. Arrows point to the position of the parasegment boundary and the two possible positions of the segmental boundary. Expression of *Wnt* genes is indicated by coloured bars. Positions of expression of the segment polarity genes *engrailed* (*en*) and *hedgehog* (*hh*) are indicated by dark grey (co-expression of *en* + *hh*) and light grey (expression of *en*) bars. Drawing is out of scale. PSB, parasegment boundary; SB?, possible position of segmental boundary; sg, salivary gland.

We observed that several onychophoran *Wnt* genes are expressed in typical segment polarity gene patterns (that is, in transverse segmental stripes), but that the intrasegmental position of these genes differs considerably (Figure 13): Three *Wnt* genes pattern the anterior (*Wnt5*), the median (*Wnt16*) and the posterior (*Wnt4*) region of each trunk segment, respectively (as indicated by the position of the limbs and the juxtaposed expression of *en/hh* and *wg*). Four onychophoran *Wnt* genes (*wg/Wnt1*, *Wnt6*, *Wnt9* and *Wnt11*) are expressed within the *Wnt16* domain in the centre of the onychophoran segments, and *Wnt10* appears to be expressed posteriorly, adjacent to the *wg/Wnt1* domain (Figure 13).

The onychophoran segment polarity gene orthologues are first expressed in their typical pattern in adjacent transverse stripes at around stage 10 [17,18]; note that *en* is expressed considerably earlier. However, most of the *Wnt* genes are expressed in segment polarity gene-like patterns only at later stages. The temporal delay of segmental patterning in comparison with segment polarity genes, such as *en*, *wg/Wnt1* and *hh*[17,18], suggests that onychophoran *Wnt* genes are not generally involved in the determination of (morphologically invisible) intersegmental boundaries, but that their function is rather restricted to intrasegmental patterning.

All onychophoran segments are added from the posterior pit region, the SAZ. Since some of the *Wnt* genes are expressed in the SAZ, these genes may be involved in segment addition. The involvement of *Wnt* genes during segmentation has been demonstrated by the knocking down of components of the canonical *Wnt* gene network in insects [37-41]. In the basally branching insect *Periplaneta americana*, and in the spider *Parasteatoda tepidariorum*, the function of *wg/Wnt1* and *Wnt8*, respectively, are indeed crucial for posterior segment addition [42-44]. In *E. kanangrensis*, we find that all *Wnt* genes except *Wnt6* and *Wnt7* are expressed in the SAZ. Of these, *wg/Wnt1*[17], *Wnt2*, *Wnt4*, *Wnt5*, *Wnt11* and *Wnt16* are all expressed prominently in the SAZ. Others, such as *Wnt9*, *Wnt10* and *WntA* are only weakly expressed in the SAZ, or are expressed ubiquitously at some stages and thus also in the SAZ. Overall, these expression patterns suggest a function of onychophoran *Wnt* genes during segment addition, similar to that reported for insects and a spider.

### Conserved and diverged aspects of *Wnt* expression in arthropods and onychophorans

*Wnt1/wg* is the best studied arthropod *Wnt* gene (reviewed in [45,46]). Its expression in transverse segmental stripes anterior adjacent to *engrailed* (*en*) is principally conserved in all arthropods [12,11,14], and even in *E. kanangrensis*[17,18]. The segment polarity gene-like function, however, has only been directly demonstrated for some insects [38,39,47].

Expression of *Wnt2* has been studied in the millipede *Glomeris marginata*, the centipede *Strigamia maritima* and the spider *P. tepidariorum*[20,48,49]. Expression in the ocular region is conserved between *E. kanangrensis* and these arthropods. However, comparable expression in an early gap-gene-like domain, at the posterior end of the embryo, and in the frontal appendages of *E. kanangrensis* is not found in these arthropods.

Expression of *Wnt4* has been studied in *G. marginata*, *S. maritima* and *P. tepidariorum*[20,48,49]. The expression profiles of *Wnt4* in these arthropods are completely different. Expression in myriapods is observed throughout the developing embryo, whereas in *P. tepidariorum*, expression is restricted to the SAZ. None of the expression patterns of *Wnt4* in myriapods and *E. kanangrensis* is the same. Reconstruction of the ancestral arthropod expression pattern of *Wnt4* is therefore impossible based on the available data.

Expression of *Wnt5* has been investigated in a number of arthropods, including *D. melanogaster*[50,51] and *T. castaneum*[52], the spiders *C. salei*[12,53] and *P. tepidariorum*[20], and the myriapods *G. marginata*[48] and *S. maritima*[49]. *Wnt5* expression in the two spiders is virtually identical, and expression in the other arthropods is in many aspects comparable to that observed in these spiders: *Wnt5* is expressed in the ventral nervous system, including the brain, transverse segmental stripes, and the labrum and the limb primordia (and later in the limbs). Expression in the brain, the limb primordia, and in the form of segmental stripes is conserved in *E. kanangrensis*. This strongly suggests an ancestral and conserved function for *Wnt5* in the development of these tissues. This assumption is supported by the fact that *Wnt5* is the only *Wnt* gene (besides *wg/Wnt1*) that is present in all hitherto studied arthropods (summarized in [20]; Figure 2). However, apparently lineage-specific expression of *Wnt5* includes the expression observed in the heart of spiders and the early gap-gene-like expression in the onychophoran.

*Wnt6* expression has been studied in *D. melanogaster*[54], *T. castaneum*[52], *G. marginata*[20] and *S. maritima*[49]. This work has shown that expression in the limbs and brain, and the segmental expression (probably associated with the central nervous system), is conserved among these arthropods. In *E. kanangrensis*, expression in the ventral nervous system and the limbs appears to be conserved, and may thus represent part of the ancestral expression pattern of *Wnt6*.

The embryonic expression of *Wnt7* has been studied in *D. melanogaster*[50,55], *T. castaneum*[52], *G. marginata*[20], *P. tepidariorum*[20] and *S. maritima*[56,49]. In both insects, *Wnt7* is expressed in a segmentally reiterated pattern; a comparable pattern is seen in *S. maritima* but not *G. marginata*. One of the two *Wnt7* paralogues of *P. tepidariorum* is expressed in the SAZ, and this pattern is also seen in *G. marginata*, but not in the onychophoran, or in *T. castaneum*. In both myriapods, *Wnt7* is expressed in the heart, and in the brain, but expression in the labrum is only seen in *G. marginata*, and expression in the antennae is only present in *S. maritima*. In summary, while some aspects of the expression of arthropod *Wnt7* genes are conserved, none of these expression domains is observed in *E. kanangrensis*.

Expression of *Wnt8* has been studied in *D. melanogaster*[57,58], where it is called *WntD*, and in *T. castaneum*[52], as well as in *G. marginata*[20] and *P. tepidariorum*[42]. In *D. melanogaster*, it is first expressed at both poles of the early blastoderm stage embryo. Later during ontogenesis, it is expressed in the mesectoderm and the ventral neurectoderm. In *T. castaneum*, *Wnt8* is only expressed at the posterior pole in blastoderm stage embryos and in the ventral mesoderm in the SAZ. The early expression at the posterior pole and in the SAZ is also conserved in the spider, and functional studies have shown that *Wnt8* is involved in posterior segment addition in both *T. castaneum* and *P. tepidariorum*[39,42]. The fact that *Wnt8* is only expressed in the primordia of the ocular region and the mandibular segment of *G. marginata* was therefore unexpected [20], and contradicted the idea that *Wnt8* could possibly play an ancestral and conserved role in arthropod segmentation [43].

We did not recover an *E. kanangrensis Wnt8* in our surveys. It may be that *Wnt8* was missed because it is expressed at a low level or because it is not expressed at all during ontogenesis. The possible lack, or nonexpression during ontogenesis, of onychophoran *Wnt8*, however, supports the possibility that *Wnt8* is not an ancestral component of the segmentation machinery of arthropods and onychophorans.

Expression of *Wnt9* has been studied in *D. melanogaster*[59], *T. castaneum*[52,49] and *G. marginata*[48]. In *T. castaneum* and *S. maritima*, *Wnt9* is only expressed in a few cells in the gut. In *G. marginata*, this gene is transiently expressed in segment polarity gene-like segmental stripes, in the appendages including the labrum, and in the SAZ. Later, it is expressed in a dorsal segmental pattern and in the form of stripes in the dorsal extraembryonic tissue. *D. melanogaster Wnt9* is also expressed in a segment polarity-like pattern and in the labrum. In *E. kanangrensis*, at least, expression in the tips of the appendages and the segment polarity gene-like expression are conserved, and this may indeed represent the ancestral expression profile of *Wnt9*.

*Wnt10* expression has so far only been studied in *D. melanogaster*[54], *S. maritima*[49] and *T. castaneum*[52]. In *D. melanogaster*, it is expressed in the mesoderm, the developing gut and the central nervous system. In *T. castaneum*, it is expressed in the cephalic lobes, the appendages and in transverse segmental stripes anteriorly adjacent to the expression of *engrailed* (*en*). This expression is also conserved in *S. maritima*. Segmental expression and expression in the limbs (discussed later) appears to be conserved between *T. castaneum, S. maritima* and the onychophoran, and may thus represent part of the ancestral expression pattern of *Wnt10*. This scenario would mean that the expression profile of *D. melanogaster Wnt10*, however, is derived.

*Wnt11* orthologues have been isolated and their expression investigated in *T. castaneum*[52], *P. tepidariorum*[20], *G. marginata*[20], and *S. maritima*[56,49]. In *P. tepidariorum*, there are two paralogues of *Wnt11*, but only one, *Wnt11-2*, is expressed in embryos. The expression profile of *Wnt11* is similar in the onychophoran, the spider and the myriapods. In all species, *Wnt11* is first expressed in the SAZ at the posterior region of the embryo. Later, expression appears in the tips of all appendages (except for *S. maritima*). However, segment polarity gene-like stripes of expression are only seen in *E. kanangrensis*. Only in *T. castaneum* is *Wnt11* expressed in the heart.

*Wnt11* is likely to play a conserved role during limb development as represented by the strong expression in the tips of the limbs, and segment addition, as represented by the strong expression in the posterior end of the embryos.

Expression of *Wnt16* has been described in *P. tepidariorum*[20], *S. maritima*[49] and *G. marginata*[14,20] (in 2004, erroneously described as *Wnt7*). In both the spider and the myriapods, *Wnt16* is expressed in transverse segmental stripes anterior and directly adjacent to the expression of *en* (somewhat unclear for *S. maritima*) suggesting that it is involved in segmental boundary formation. *Wnt16* is also expressed in the developing brain and in the tips and ventral tissue of the limbs. The arthropod *Wnt16*-expression profile is conserved in *E. kanangrensis*. One important difference is, however, that the segmental expression of *Wnt16* in *E. kanangrensis* reaches posteriorly into the domain of *en* expression.

Expression of *WntA* has been analyzed in *T. castaneum*[52], *S. maritima*[49] and *G. marginata*[14,20] (in 2004, erroneously described as *Wnt5*), and *C. salei*[20]. Expression in the mandibulate arthropods is comparable. It is strongly expressed in the developing brain, heart, limbs and central nervous system. In *E. kanangrensis*, *WntA* is similarly expressed in the brain (head lobe), the limbs and, at later stages, in the ventral nervous system; expression in the heart, however, is not observed in the onychophoran. Expression of *WntA* in the spider *C. salei* differs significantly from that in the other arthropods and the onychophoran. The only possibly conserved pattern of *WntA* is in the SAZ (apart from that, spider *WntA* is only expressed in small domains in the spinnerets and the chelicerae).

### The onychophoran ‘segment’

Onychophorans represent segmented animals, although some of the key characteristics of the arthropods, such as full adult body segmentation with pronounced segmental indentations are not present. The latter is best interpreted as being primitive [60]. Since segmental indentations are lacking in onychophorans, it is difficult to determine the position of the segmental boundaries in the ectoderm. The best approximation may be given by the expression of segment polarity genes that determine segmental and parasegmental boundaries in arthropods [12,61].

The segment polarity gene network is conserved in arthropods (for example, [11,12,14,62]) and onychophorans [17,18]. The parasegmental boundary in arthropods lies at the interface between *en* and *wg* expressing cells, and the segmental boundary lies posterior to the expression of *en* (for example, [12,63]). This means that the parasegmental boundaries of onychophorans are located in the posterior of the limbs, exactly as in arthropods. Determination of the segmental boundaries by means of gene expression patterns is not that clear because the domain of *en* expression is broadened in ventral tissue [17] and the posterior border of each stripe thus lies posterior to that of *hh*[18], which is the direct downstream target of *en* in arthropods [15,64]. Therefore, the segmental boundaries lie either posterior to the *en/hh* domain and thus directly posterior to the limbs, or the segmental boundaries lie posterior to *en* and thus somewhat shifted towards the posterior (compare with Figure 13).

### *Wnt* genes and limbs

**Figure 14:**
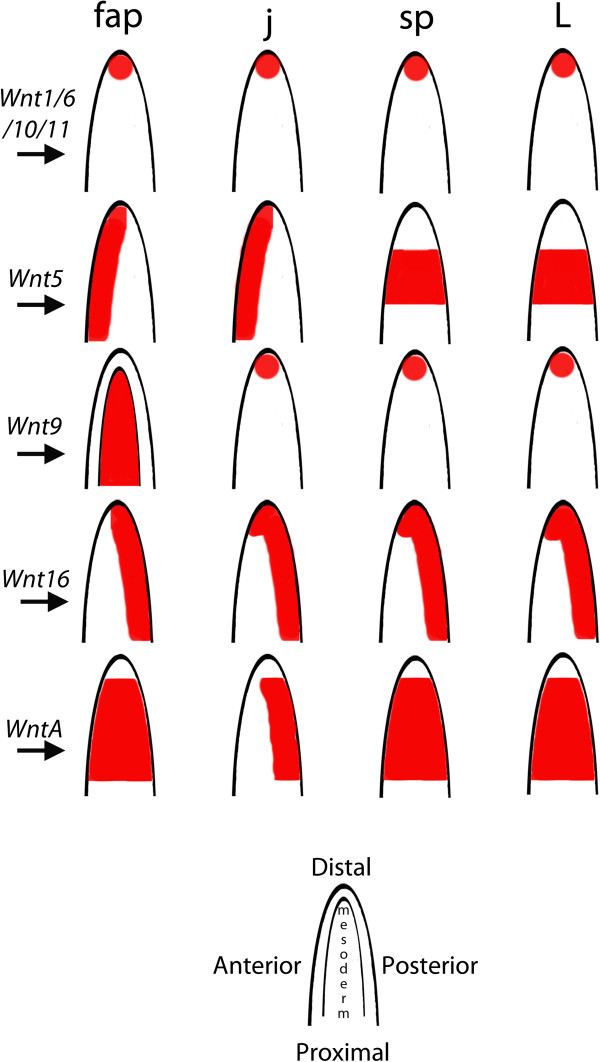
**Summary of the expression of *E. kanangrensis Wnt *genes in the appendages.** Expression of *Wnt* genes in the limbs is shown in red. Expression is ectodermal, if not indicated differently (that is, *Wnt9* in the frontal appendages is mesodermal). Note that mesodermal expression of *Wnt4* in the frontal appendages and the jaws is not shown.

Comparative analyses of *Wnt* gene expression in a wide range of arthropods indicates that they play an important role in limb development. Typically, *Wnt* genes are expressed along the ventral side of the limbs [14,20,48,52,65,66]. It was, therefore, proposed that Wnt patterning could have a conserved and potentially combinatorial function in ventral limb patterning in arthropods, acting upstream of ventralizing limb genes, such as *midline/H15* (*mid/H15*) [41,65,67-70] (but see [38] for a different opinion on the possibly conserved function of *wg/Wnt1* in ventral limb development). The expression of *wg/Wnt1* in the tips of the limbs in *E. kanangrensis* was thus somewhat unexpected [17]. This could have been explained by the potential presence of additional, but at the time unstudied, *Wnt* genes that could substitute for *wg/Wnt1* in ventral tissue of the onychophoran limbs. Our comprehensive data on onychophoran *Wnt* gene expression now reveals that the expression of *wg/Wnt1* in the tips of the limbs does not represent an exception. In arthropods, not only *wg/Wnt1* but also a number of other *Wnt* genes are expressed along the ventral side of the limbs [20,48,52]. Recent studies suggest that *Wnt* genes and their receptors may act in combination or may have redundant functions, especially during segmentation and limb development, which would explain the relatively high number of *Wnt* genes with identical expression patterns in the limbs [41,52]. In the onychophoran, five *Wnt* genes are expressed in the tips of at least a subset of appendages (summarized in Figure 14). This implies a general change of expression from ‘all-ventral’ to ‘distal-only’. Whether this shift occurred in the lineage leading to the arthropods or whether it comprises an ancestral feature that has changed in the evolutionary line represented by onychophorans remains unclear and data from an outgroup, such as tardigrades, is required.

Interestingly, a recent study revealed two separate functions of *wg/Wnt1* in limb development in *T. castaneum*[70]. One function concerns ventral limb patterning; the other concerns the proximodistal growth of the limbs. It is therefore likely that the expression seen in onychophorans is associated with limb growth, rather than with a function in dorsoventral patterning. This would mean that at least this component of dorsoventral patterning evolved in the lineage leading to the arthropods. The evolution of the dual function of *Wnt* genes during limb development may be reflected by the situation in the millipede *G. marginata*, where some *Wnt* genes are expressed strongly in the tips of the limbs, but only weakly along the ventral side of the limbs [48]. This implies that the expression in the tips as seen in the onychophoran may represent the ancestral state and that ventral expression evolved step by step within the arthropod lineage.

## Conclusions

Wnt ligands play important roles during animal development. Our study shows that most of the protostome Wnt ligands are present in onychophorans, and that all of those present are expressed in distinct patterns throughout embryogenesis. It is surprising that eight of twelve *Wnt* genes are expressed in segmental stripes reminiscent of the expression of classical arthropod segment polarity genes, and that their combined expression covers the complete segments. This suggests that *Wnt* genes may play a more prominent role in segment regionalization than they do in arthropods, where the expression of *Wnt* genes is mostly restricted to anterior cells abutting the domain of *engrailed* (*en*) expression. Early expression of *Wnt2*, *Wnt4* and *Wnt5* in gap-gene-like and *Hox*-gene-like patterns suggests a contributing role of these genes in giving anterior segments their specific identities. Strong expression of most of the onychophoran *Wnt* genes in the posterior SAZ might be correlated with a role in germ band elongation or segmentation. Thus, it seems likely that *Wnt* genes are involved in segment formation, segment regionalization and the definition of segment identity in onychophorans. If these assumptions hold true, the role of *Wnt* genes in onychophoran segmentation would clearly extend their roles in arthropod segmentation.

## Abbreviations

DAPI: 4-6-diamidino-2-phenylindole; MCMCMC: metropolis-coupled Markov chain Monte Carlo; PBST: phosphate-buffered saline with 0.1% Tween-20; PCR: polymerase chain reaction; SAZ: segment addition zone.

## Competing interests

The authors declare that they have no competing interests.

## Authors’ contributions

MH carried out most of the experiments, discussed the experimental outline and wrote part of the first draft of the manuscript. AS was involved in performing the phylogenetic analysis. GEB discussed the experimental outline, was involved in drafting the final version of the manuscript and initiated work on onychophoran segmentation. NP was involved in performing the phylogenetic analysis, assembled the *E. kanangrensis* transcriptome and was involved in drafting the final version of the manuscript. APM was involved in performing the phylogenetic analysis and was involved in drafting the final version of the manuscript. RJ was mainly responsible for the experimental outline, carried out part of the experiments, wrote part of the first draft of the manuscript and was involved in drafting the final version of the manuscript. All authors read and approved the final manuscript.

## Supplementary Material

Additional file 1: Figure S1 Schematic summary of early multiple-segment-wide expression domains of *Wnt* genes compared with the expression of *Hox* genes. This figure has been modified after [26]. Expression of *Wnt* genes is indicated by black, dark grey and light grey bars. Low level expression is indicated by thin bars and ‘w’. 1–15, first to fifteenth leg-bearing segments; *abd-A*, *abdominal-A*, *Abd-B*, *Abdominal-B*; *Antp*, *Antennapedia*; *Dfd*, *Deformed*; fap, frontal appendage; *ftz*, *fushi-tarazu*; hl, head lobe; j, jaw; *lab*, *labial*; *pb*, *proboscipedia*; SAZ, segment addition zone; *Scr*, *Sex combs reduced*; sp, slime papilla; *Ubx*, *Ultrabithorax*. Click here for file
