# Supplementary material for: Analysis of the Wnt gene repertoire in an onychophoran provides new insights into the evolution of segmentation
Source: EvoDevo. 2014 Apr 3;5:14. doi: 10.1186/2041-9139-5-14 (PMC4021614; doi:10.1186/2041-9139-5-14)
Supplement: Additional file 1: Figure S1 — Schematic summary of early multiple-segment-wide expression domains of Wnt genes compared with the expression of Hox genes. This figure has been modified after [26]. Expression of Wnt genes is indicated by black, dark grey and light grey bars. Low level expression is indicated by thin bars and ‘w’. 1–15, first to fifteenth leg-bearing segments; abd-A, abdominal-A, Abd-B, Abdominal-B; Antp, Antennapedia; Dfd, Deformed; fap, frontal appendage; ftz, fushi-tarazu; hl, head lobe; j, jaw; lab, labial; pb, proboscipedia; SAZ, segment addition zone; Scr, Sex combs reduced; sp, slime papilla; Ubx, Ultrabithorax. [file 2041-9139-5-14-S1.pdf]

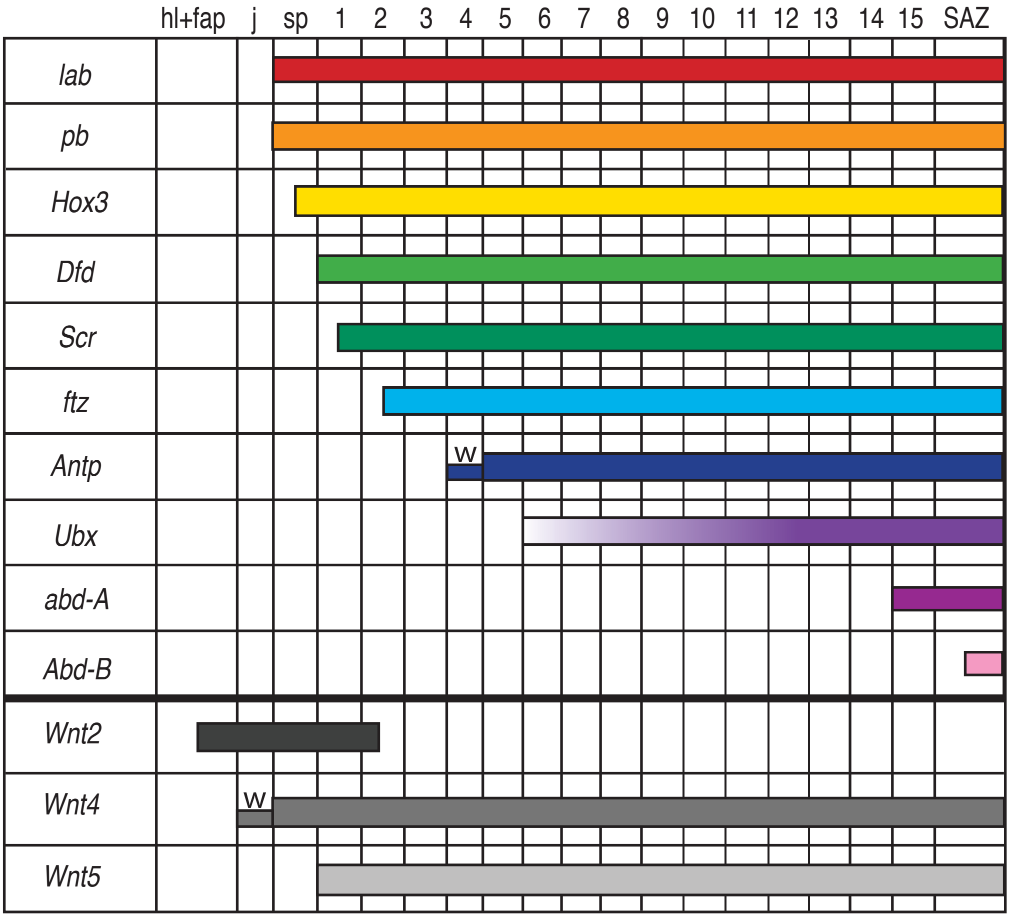
hl+fap
j
sp
1
2
3
4
5
6
7
8
9
10
11
12
13
14
15
SAZ
lab
pb
Hox3
Dfd
Scr
ftz
Antp
w
Ubx
abd-A
Abd-B
Wnt2
Wnt4
w
Wnt5
